# Prognostic factors and nomogram for pulmonary resected high-grade neuroendocrine carcinomas: a 20-year single institutional real-world experience

**DOI:** 10.1186/s13023-024-03240-8

**Published:** 2024-06-12

**Authors:** Lei Liu, Jiaqi Zhang, Ke Zhao, Chao Guo, Cheng Huang, Shanqing Li, Yeye Chen

**Affiliations:** Department of Thoracic Surgery, Peking Union Medical College Hospital, Chinese Academy of Medical Sciences & Peking Union Medical College, No.1 Shuaifuyuan, Wangfujing Street, Dongcheng District, Beijing 100730 P. R. China

**Keywords:** Pulmonary high-grade neuroendocrine carcinoma, Prognostic factors, Nomogram, Surgical resection, Overall survival

## Abstract

**Background:**

Pulmonary high-grade neuroendocrine carcinomas(pHGNEC) encompassing small cell lung cancer (SCLC) and large cell neuroendocrine carcinoma (LCNEC) are clinically aggressive tumors with poor prognosis. The role of surgery and prognostic factors guiding management remain unclear. We aimed to analyze prognosis following resection and identify predictive variables.

**Methods:**

This retrospective study analyzed 259 patients undergoing pHGNEC resection from 2001–2023. Overall survival (OS) and disease-free survival (DFS) were evaluated using Kaplan–Meier curves. Prognostic factors were assessed with Cox regression and visualized using nomogram tools.

**Results:**

Minimally invasive surgery was associated with better OS (*p* = 0.001) and DFS (*p* = 0.001). Higher T stage predicted worse OS (T2 *p* = 0.044, T4 *p* = 0.007) and DFS (T2 *p* = 0.020, T4 *p* = 0.004). Advanced TNM stage III (OS *p* = 0.018; DFS *p* = 0.015) and IV (OS *p* < 0.001; DFS *p* < 0.001) also correlated with poorer prognosis. In the SCLC subgroup, elevated preoperative CEA independently predicted worse OS (*p* = 0.012) and DFS (*p* = 0.004). T4 disease (OS *p* < 0.001; DFS *p* = 0.002) and advanced TNM staging (stage III OS *p* = 0.043; DFS *p* = 0.045; stage IV OS *p* < 0.001, DFS *p* < 0.001) were associated with worse outcomes. In LCNEC patients, VATS resection improved OS (*p* = 0.048) and DFS (*p* = 0.027) despite conversion. Prior malignancy predicted worse OS (*p* < 0.001). Advanced TNM disease (stage III OS *p* = 0.047; stage IV OS *p* = 0.003, DFS *p* = 0.005) were also negative prognostic factors. The prognostic nomogram incorporating above variables effectively stratified risk. Calibration plots revealed good correlation between predicted and actual survival.

**Conclusions:**

We identified minimally invasive surgery, early TNM stage, younger age, and normal preoperative CEA as positive prognostic factors following pHGNEC resection. Our study provides an applicable prognostic nomogram to facilitate personalized pHGNEC management.

## Background

Pulmonary high-grade neuroendocrine carcinoma (pHGNEC) is a rare and aggressive group of lung cancers that encompass both small cell lung cancer (SCLC) and large cell neuroendocrine carcinoma (LCNEC). Due to their rapidly progressive nature and high metastatic potential, the prognosis for patients diagnosed with these tumors is generally poor [1, 2].

The incidence of pHGNECs has been increasing, reflecting the need for improved diagnostic and treatment strategies [3]. Surgical resection is the primary treatment modality for early-stage disease, aiming for complete tumor excision and potential cure. However, the majority of patients with pHGNECs present with advanced disease at the time of diagnosis, which often precludes curative surgery and necessitates a multimodal approach, including chemotherapy, radiation therapy, and targeted therapies [4]. Recent advances in surgical interventions, targeted therapy, and immunotherapy have shown promising results in the management of pHGNECs [5–7]. Several recent studies have demonstrated improvements in survival outcomes for patients with early-stage pHGNECs who have undergone surgical resection, indicating that surgery could play a vital role in the management of these tumors [8–10].

This retrospective study analyzed patients with pHGNEC who underwent surgery at our center. Long-term follow-up prognostic data were included to conduct the analysis. A prognostic prediction model was constructed using a nomogram in order to provide references for individualized treatment and prognostic prediction for this group of patients going forward.

## Methods

### Patient population

This retrospective study analyzed data from patients who underwent surgical treatment for pathologically diagnosed pHGNECs at our center between March 2001 and May 2023. Parameters analyzed included age, sex, smoking history, surgical methods, pathological subtypes, lymph node dissection, postoperative adjuvant treatment, survival status, tumor metastasis status, and other relevant factors. All patients were restaged according to the 8th edition of the UICC-TNM classification system. This study was performed with authorization from the Institutional Review Board of Peking Union Medical College Hospital, Beijing, China (S-K2117).

### Follow-up

After surgery, patients underwent routine surveillance including physical examinations, blood tests, and chest CT scans every 3–6 months for 5 years. Additional testing such as bone scans, head enhanced MRI, and PET/CT scans were conducted annually to monitor for distant metastases. Patients underwent full systemic workups if any concerning symptoms or signs appeared. Overall survival (OS) was defined as the time from surgical resection to death. Disease-free survival (DFS) was the time from surgery until locoregional recurrence, distant recurrence, or death from any cause.

### Statistical analysis

Nomograms were constructed to model patient survival using a 3-step process: a. Unadjusted univariate Cox regression analyzed prognostic risks for OS and DFS; b. Significant variables (*p* < 0.05) were entered into a multivariate Cox proportional hazards model to calculate hazard ratios (HR) and 95% confidence interval (CI); c. Variables that remained statistically significant (*p* < 0.05) in the Cox model were incorporated to build the nomograms. The final nomogram models integrated all predictive features to estimate survival probabilities.

Kaplan–Meier curves depicted OS and DFS, with log-rank tests to compare groups stratified by nomogram signatures.

The nomogram predicted-1, 3- and 5-year OS and DFS rates. Due to incomplete external data variables, only internal verification was performed for the nomogram, and the discrimination and calibration of the model were evaluated. The evaluation of discrimination in this article was based on the index of concordance (C-index). The closer the C-index was to 1, the better the predicted results of the model. Evaluation of the degree of calibration was based on the calibration plot method, which involved a comparison between the event incidence predicted by the nomogram model and the true incidence. All analyses were performed in R software (version 3.3.3).

## Results

### General information

Within the time frame of this study, a total of 303 patients with pHGNEC received surgical-related treatment at our center. Excluding 11 patients who were lost to follow-up and 33 patients only underwent biopsy surgery, a total of 259 patients were enrolled in this study, including 205 male patients and 54 female patients. The average age of the enrolled patients was 60.5 years (range 30–83 years), and the median follow-up time was 32 months (range 1–239 months). By the time of the last follow up, 121 patients were still alive. Among the enrolled patients, there were 146 patients with SCLC, 78 patients with LCNEC, and 35 patients with mixed type tumors (Table 1). The interquartile ranges of intraoperative blood loss for patients with pHGNEC, SCLC, and LCNEC were 200 ml, 300 ml, and 250 ml, respectively. Among the enrolled patients, 75 patients underwent preoperative bronchoscopy or puncture biopsy, with 46 cases (61.33%) diagnosed as malignant tumors, but only 34 cases (45.33%) had consistent pathological results between the biopsy and postoperative pathology. Intraoperative frozen section examination was performed in 169 patients, but only 81 cases (47.92%) were suggested to have SCLC or neuroendocrine tumors.

**Table 1:** Characteristics of all the enrolled pHGNEC patients

Variable	N (%)
Gender	
Male	205 (79.1%)
Female	54 (20.9%)
Mean age (years)	60.51 (range 30–83)
Median follow-up time (months)	32 (range 1–239)
Smoking History	
Yes	178 (68.7%)
No	81 (31.3%)
Tumor location	
Central	105 (40.5%)
Peripheral	147 (56.8%)
Indistinguishable	7 (2.7%)
Neoadjuvant chemotherapy	
Yes	44 (17.0%)
No	215 (83.0%)
Adjuvant chemotherapy	
Yes	222 (85.7%)
No	37 (14.3%)
Adjuvant radiotherapy	
Yes	63 (24.3%)
No	196 (75.7%)
Pathology Subtype	
Small cell lung cancer (SCLC)	146 (56.4%)
Large cell neuroendocrine carcinoma (LCNEC)	78 (30.1%)
Mix^a^	35 (13.5%)
Approach	
Thoracotomy	62 (23.9%)
VATS	182 (70.3%)
VATS converted to thoracotomy	15 (5.8%)
Resection extent	
Lobectomy	183 (70.7%)
Combined lobectomy	52 (20.1%)
Sublobectomy	24 (9.2%)
T	
1	71 (27.4%)
2	134 (51.7)
3	39 (15.1%)
4	15 (5.8%)
N	
0	122 (47.1%)
1	60 (23.2%)
2	77 (29.7%)
M	
0	244 (94.2)
1	12 (4.6%)
2	3 (1.2%)
TNM	
I	99 (38.2%)
II	50 (19.3%)
III	95 (36.7%)
IV	15 (5.8%)

^a^including: SCLC + squamous cell carcinoma (SCC), LCNEC + adenocarcinoma, LCNEC + SCLC, SCLC + adenocarcinoma, SCLC + atypical carcinoid (ATC), LCNEC + SCLC + adenocarcinoma, LCNEC + SCC

### Univariable analysis of prognostic factors

In the univariate analysis for DFS, several key factors emerged as significant prognostic indicators. Patients with larger maximal tumor diameters exhibited poorer outcomes (HR 1.25, 95% CI 1.13–1.37, *p* < 0.001). Correspondingly, higher tumor stages, both in terms of primary tumor extent (T), nodal involvement (N), distant metastasis (M), and the composite TNM staging, were consistently associated with diminished DFS. Surgical approach also played a role, with patients treated via video-assisted thoracic surgery (VATS) demonstrating markedly improved prognoses compared to thoracotomy (HR 0.53, 95% CI 0.36–0.77, *p* = 0.001).

**Fig. 1:**
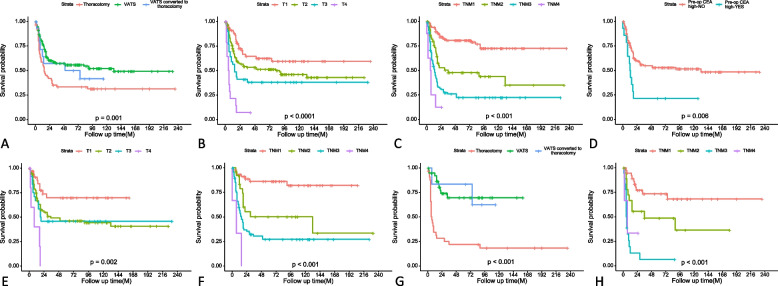
Kaplan–Meier curves of DFS for patients with pHGNEC, SCLC and LCNEC: pHGNEC: **A** resection extent; **B** T stage; **C** TNM stage; SCLC: **D** pre-op CEA high; **E** T stage; **F** TNM stage; LCNEC: **G** resection extent; **H** TNM stage

Subgroup analysis of SCLC patients corroborated the detrimental impact of advanced T, N, and TNM stages. Notably, elevated preoperative carcinoembryonic antigen (CEA) levels independently predicted worse DFS (HR 2.50, 95% CI 1.31–4.79, *p* = 0.006). In the LCNEC subgroup, adjuvant radiotherapy was associated with relatively poorer DFS (HR 2.60, 95% CI 1.08–6.26, *p* = 0.033). However, peripheral tumor distribution (HR 0.39, 95% CI 0.20–0.75, *p* = 0.005) and VATS approach, even when converted to open thoracotomy (HR 0.22, 95% CI 0.05–0.91, *p* = 0.037), conferred significant prognostic benefits. Regarding the extent of surgical resection, complex or combined lobectomies were associated with worse outcomes compared to standard lobectomy or sublobar resection (HR 2.48, 95% CI 1.11–5.53, *p* = 0.026). Consistent with the SCLC findings, advanced T, N, and TNM stages predicted poorer DFS in the LCNEC subgroup. The Kaplan–Meier curves illustrating these DFS results are presented in Fig. 1.

Turning to overall survival (OS), increasing age (HR 1.02 per year, 95% CI 1.00–1.04, *p* = 0.022) and the history of other malignancies (HR 2.17, 95% CI 1.06–4.47, *p* = 0.035) were associated with poorer prognosis. As with DFS, larger tumor diameters significantly predicted worse OS outcomes (HR 1.25, 95% CI 1.13–1.37, *p* < 0.001). Surgical approach continued to play a pivotal role, with VATS associated with longer OS compared to open thoracotomy (HR 0.58, 95% CI 0.40–0.84, *p* = 0.004). Advancing T, N, and TNM stages corresponded with diminished OS in the overall cohort.

**Fig. 2:**
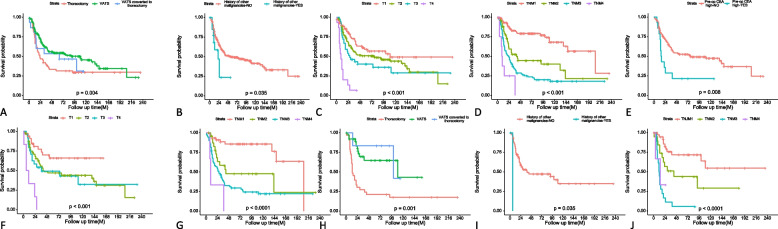
Kaplan–Meier curves of OS for patients with pHGNEC, SCLC and LCNEC: pHGNEC: **A** resection extent; **B** history of other malignancies; **C** T stage; SCLC: **D** TNM stage; SCLC: **E** pre-op CEA high; **F** T stage; **G** TNM stage; LCNEC: **H** resection extent; **I** history of other malignancies; **J** TNM stage

Within the SCLC subgroup, larger tumor size emerged as an independent adverse prognostic factor (HR 1.18 per cm, 95% CI 1.01–1.38, *p* = 0.032), while elevated preoperative CEA levels conferred a worse OS (HR 2.40, 95% CI 1.26–4.5, *p* = 0.008), consistent with the DFS findings. In the LCNEC subgroup, history of other malignancies were associated with a increase in the risk of death (HR 23.51, 95% CI 2.33–236.96, *p* = 0.007), and patients with endocrine comorbidities also exhibited significantly poorer OS (HR 2.50, 95% CI 1.14–5.47, *p* = 0.022). Mirroring the DFS results, VATS approach, even when converted to thoracotomy (HR 0.22, 95% CI 0.05–0.94, *p* = 0.041), conferred substantial OS benefits. Regarding tumor location, central tumors predicted improved OS compared to peripheral lesions (HR 0.44, 95% CI 0.23–0.83, *p* = 0.011). Both SCLC and LCNEC subgroup analyses reaffirmed the profound adverse prognostic impact of advancing T, N, and TNM stages. The Kaplan–Meier curves illustrating these OS results are presented in Fig. 2.

### Multivariable analysis of prognostic factors

In the multivariate analysis for DFS, patients who underwent VATS surgery exhibited substantially better outcomes compared to those treated via thoracotomy(HR 0.50, 95% CI 0.33–0.75, *p* = 0.001). Advancing T stage was associated with diminished DFS. Patients with T2 (HR 1.84, 95% CI 1.10–3.06, *p* = 0.020) and T4 (HR 3.76, 95% CI 1.53–9.24, *p* = 0.004) lesions demonstrated worse prognosis respectively. Similarly, advancing composite TNM stage emerged as a potent adverse prognostic factor (Table 2).

**Table 2:** Univariable and multivariable analyses of disease-free survival and overall survival for all the enrolled pHGNEC patients

Variable		N(%)	DFS		OS	
			HR (univariable)	HR (multivariable)	HR (univariable)	HR (multivariable)
Gender	Male	205 (79.2%)				
	Female	54 (20.8%)	0.76 (0.48–1.20, *p* = .237)		0.71 (0.45–1.11, *p* = .137)	
Age (years)	Mean ± SD	60.5 ± 9.6	1.02 (1.00–1.04, *p* = .115)		1.02 (1.00–1.04, *p* = .022)	1.03 (1.01–1.06, *p* = .002)
Neoadjuvant chemotherapy	No	215 (83.0%)				
	Yes	44 (17.0%)	1.19 (0.76–1.86, *p* = .442)		1.14 (0.75–1.74, *p* = .546)	
Adjuvant chemotherapy	No	37 (14.3%)				
	Yes	222 (85.7%)	1.43 (0.80–2.53, *p* = .226)		1.12 (0.68–1.87, *p* = .654)	
Adjuvant radiotherapy	No	196 (75.7%)				
	Yes	63 (24.3%)	1.32 (0.90–1.94, *p* = .155)		1.16 (0.80–1.69, *p* = .425)	
Duration of disease (months)	Mean ± SD	4.4 ± 6.0	1.00 (0.98–1.03, *p* = .754)		1.00 (0.97–1.03, *p* = .892)	
Symptoms	No	129 (49.8%)				
	Yes	130 (50.2%)	1.11 (0.78–1.57, *p* = .574)		0.96 (0.69–1.35, *p* = .826)	
Comorbid neuroendocrine symptoms	No	250 (96.5%)				
	Yes	9 (3.5%)	1.04 (0.42–2.54, *p* = .938)		1.04 (0.42–2.53, *p* = .939)	
History of other malignancies	No	245 (94.6%)				
	Yes	14 (5.4%)	1.59 (0.74–3.42, *p* = .237)		2.17 (1.06–4.47, *p* = .035)	1.91 (0.88–4.15, *p* = .101)
Comorbid cardiovascular diseases	No	156 (60.2%)				
	Yes	103 (39.8%)	0.78 (0.54–1.13, *p* = .192)		0.95 (0.67–1.35, *p* = .782)	
Comorbid neurological diseases	No	248 (95.8%)				
	Yes	11 (4.2%)	1.03 (0.46–2.35, *p* = .935)		1.11 (0.52–2.37, *p* = .791)	
Comorbid endocrine diseases	No	223 (86.1%)				
	Yes	36 (13.9%)	1.24 (0.76–2.02, *p* = .383)		1.33 (0.84–2.12, *p* = .230)	
Comorbid respiratory diseases	No	234 (90.3%)				
	Yes	25 (9.7%)	0.83 (0.44–1.59, *p* = .580)		1.13 (0.64–2.01, *p* = .671)	
Tumor location	Central	105 (40.5%)				
	Peripheral	147 (56.8%)	0.80 (0.56–1.14, *p* = .220)		0.91 (0.65–1.28, *p* = .580)	
	Indistinguishable	7 (2.7%)	0.26 (0.04–1.84, *p* = .176)		0.29 (0.04–2.11, *p* = .222)	
Max diameter (cm)	Mean ± SD	3.2 ± 1.8	1.25 (1.13–1.37, *p* < .001)	0.99 (0.89–1.11, *p* = .866)	1.25 (1.13–1.37, *p* < .001)	1.03 (0.93–1.15, *p* = .569)
Smoking history	No	81 (31.3%)				
	Yes	178 (68.7%)	1.22 (0.83–1.81, *p* = .308)		1.34 (0.92–1.95, *p* = .131)	
Family history	No	219 (84.6%)				
	Yes	40 (15.4%)	0.88 (0.53–1.44, *p* = .603)		0.93 (0.58–1.48, *p* = .756)	
Intraoperative blood loss (ml)	Mean ± SD	315.0 ± 734.5	1.00 (1.00–1.00, *p* < .001)	1.00 (1.00–1.00, *p* = .076)	1.00 (1.00–1.00, *p* < .001)	1.00 (1.00–1.00, *p* = .014)
Blood transfusion	No	215 (83.0%)				
	Yes	44 (17.0%)	1.36 (0.88–2.09, *p* = .164)		1.27 (0.84–1.92, *p* = .265)	
Approach	Thoracotomy	62 (23.9%)				
	VATS	182 (70.3%)	0.53 (0.36–0.77, *p* = .001)	0.50 (0.33–0.75, *p* = .001)	0.58 (0.40–0.84, *p* = .004)	0.49 (0.33–0.74, *p* = .001)
	VATS converted to thoracotomy	15 (5.8%)	0.71 (0.33–1.52,* p* = .381)	0.52 (0.24–1.13, *p* = .098)	0.77 (0.38–1.59, *p* = .484)	0.59 (0.28–1.25, *p* = .170)
Resection extent	Lobectomy	183 (70.7%)				
	Combined lobectomy	52 (20.1%)	1.46 (0.96–2.23, *p* = .078)		1.38 (0.92–2.07, *p* = .124)	
	Sublobectomy	24 (9.2%)	1.21 (0.68–2.18, *p* = .517)		1.39 (0.79–2.44, *p* = .258)	
Pathologic type	SCLC	146 (56.4%)				
	LCNEC	78 (30.1%)	1.10 (0.74–1.64, *p* = .620)		1.16 (0.79–1.69, *p* = .449)	
	Mix	35 (13.5%)	1.11 (0.67–1.85, *p* = .677)		1.18 (0.72–1.91, *p* = .514)	
T stage	1	71 (27.4%)				
	2	134 (51.7%)	1.63 (1.01–2.61, *p* = .044)	1.84 (1.10–3.06, *p* = .020)	1.42 (0.91–2.20, *p* = .124)	1.64 (1.01–2.65, *p* = .044)
	3	39 (15.1%)	2.41 (1.36–4.28, *p* = .003)	1.54 (0.77–3.08, *p* = .218)	1.93 (1.12–3.32, *p* = .018)	1.09 (0.55–2.16, *p* = .802)
	4	15 (5.8%)	8.30 (4.16–16.56, *p* < .001)	3.76 (1.53–9.24, *p* = .004)	7.96 (4.11–15.40, *p* < .001)	3.20 (1.37–7.51, *p* = .007)
N stage	0	122 (47.1%)				
	1	60 (23.2%)	3.34 (2.08–5.34, *p* < .001)	1.72 (0.88–3.34, *p* = .110)	3.08 (1.97–4.83, *p* < .001)	1.80 (0.95–3.42, *p* = .071)
	2	77 (29.7%)	4.72 (3.05–7.30, *p* < .001)	1.96 (0.83–4.65, *p* = .125)	3.94 (2.61–5.96, *p* < .001)	1.72 (0.74–4.04, *p* = .210)
M stage	0	244 (94.2%)				
	1	12 (4.6%)	1.83 (0.89–3.74, *p* = .099)		1.72 (0.84–3.51, *p* = .139)	
	2	3 (1.2%)	1.59 (0.39–6.45, *p* = .514)		1.32 (0.33–5.34, *p* = .697)	
TNM stage	1	99 (38.2%)				
	2	50 (19.3%)	3.08 (1.74–5.44, *p* < .001)	1.93 (0.92–4.08, *p* = .084)	2.86 (1.68–4.89, *p* < .001)	1.76 (0.86–3.61, *p* = .123)
	3	95 (36.7%)	5.96 (3.69–9.64, *p* < .001)	3.23 (1.26–8.30, *p* = .015)	4.87 (3.11–7.61, *p* < .001)	3.02 (1.20–7.59, *p* = .018)
	4	15(5.8%)	13.96 (5.89–33.06, *p* < .001)	9.53 (3.27–27.80, *p* < .001)	11.87 (5.08–27.71, *p* < .001)	9.62 (3.56–26.00, *p* < .001)
Ki67 ≥ 60%	No	19 (11.4%)				
	Yes	147 (88.6%)	1.20 (0.58–2.51, *p* = .624)		1.18 (0.59–2.37, *p* = .637)	
Pre-op SCCAg high (> 2.7 ng/ml)	NO	254 (98.1%)				
	Yes	5 (1.9%)	0.89 (0.22–3.58, *p* = .866)		0.88 (0.22–3.54, *p* = .852)	
Pre-op CEA high (> 5.0 ng/ml)	No	221 (85.3%)				
	Yes	38 (14.7%)	1.46 (0.94–2.28, *p* = .094)		1.45 (0.93–2.25, *p* = .103)	
Pre-op NSE high (> 16.3 ng/ml)	NO	196 (75.7%)				
	Yes	63 (24.3%)	1.20 (0.81–1.78, *p* = .354)		1.19 (0.81–1.75, *p* = .387)	
Pre-op Cyfra-211 high (> 3.5 ng/ml)	NO	228 (88.0%)				
	Yes	31 (12.0%)	1.24 (0.74–2.06, *p* = .413)		1.40 (0.87–2.24, *p* = .169)	
Pre-op proGRP high (> 69.2 ng/ml)	NO	195 (75.3%)				
	Yes	64 (24.7%)	0.98 (0.65–1.48, *p* = .934)		1.03 (0.69–1.55, *p* = .870)	

Subgroup analysis of SCLC patients corroborated these findings. Advancing T stage continued to predict poorer DFS. Similarly, advancing TNM stage was associated with substantially diminished DFS in the SCLC subgroup. Furthermore, elevated preoperative CEA levels emerged as an independent adverse prognostic factor (HR 2.71, 95% CI 1.36–5.39, *p* = 0.004) (Table 3). In the LCNEC subgroup, VATS approach was associated with improved DFS compared to thoracotomy (HR 0.35, 95% CI 0.14–0.89, *p* = 0.027). This benefit was maintained even when conversion to open thoracotomy was required (HR 0.19, 95% CI 0.04–0.99, *p* = 0.049). Advancing to stage IV disease emerged as a profound adverse prognostic factor (HR 13.66, 95% CI 2.23–83.72, *p* = 0.005) (Table 4).

**Table 3:** Univariable and multivariable analyses of disease-free survival and overall survival for all the enrolled SCLC patients

Item		all	DFS		OS	
			HR (univariable)	HR (multivariable)	HR (univariable)	HR (multivariable)
Gender	Male	108 (74.0%)				
	Female	38 (26.0%)	0.80 (0.46–1.40, *p* = .442)		0.71 (0.41–1.24, *p* = .235)	
Age (years)	Mean ± SD	59.0 ± 9.8	1.02 (0.99–1.05, *p* = .157)		1.02 (1.00–1.05, *p* = .067)	
Neoadjuvant chemotherapy	No	112 (76.7%)				
	Yes	34 (23.3%)	0.96 (0.55–1.67, *p* = .874)		0.98 (0.58–1.66, *p* = .947)	
Adjuvant chemotherapy	No	14(9.59%)				
	Yes	132(90.41%)	50.00(0.00–247.67, *p* = 0.75)		53.00(0.00–110.25, *p* = 0.537)	
Adjuvant radiotherapy	No	98 (67.1%)				
	Yes	48 (32.9%)	1.34 (0.83–2.17, *p* = .236)		1.14 (0.71–1.82, *p* = .597)	
Duration of disease (months)	Mean ± SD	4.4 ± 6.6	1.00 (0.96–1.03, *p* = .939)		1.00 (0.96–1.03, *p* = .849)	
Symptoms	No	67 (45.9%)				
	Yes	79 (54.1%)	0.93 (0.58–1.49, *p* = .757)		0.75 (0.48–1.19, *p* = .224)	
Comorbid neuroendocrine symptoms	No	138 (94.5%)				
	Yes	8 (5.5%)	0.93 (0.34–2.55, *p* = .887)		0.96 (0.35–2.63, *p* = .935)	
History of other malignancies	No	135 (92.5%)				
	Yes	11 (7.5%)	1.64 (0.71–3.81, *p* = .250)		2.05 (0.88–4.78, *p* = .097)	
Comorbid cardiovascular diseases	No	90 (61.6%)				
	Yes	56 (38.4%)	0.66 (0.40–1.12, *p* = .123)		0.80 (0.49–1.30, *p* = .369)	
Comorbid neurological diseases	No	141 (96.6%)				
	Yes	5 (3.4%)	1.15 (0.36–3.66, *p* = .813)		1.48 (0.54–4.07, *p* = .443)	
Comorbid endocrine diseases	No	126 (86.3%)				
	Yes	20 (13.7%)	1.40 (0.73–2.67, *p* = .308)		1.35 (0.71–2.57, *p* = .357)	
Comorbid respiratory diseases	No	141 (96.6%)				
	Yes	5 (3.4%)	1.92 (0.60–6.13, *p* = .269)		2.60 (0.95–7.14, *p* = .064)	
Tumor location	Central	77 (52.7%)				
	Peripheral	63 (43.2%)	1.13(0.71,1.81, *p* = .612)		1.22 (0.77,1.93, *p* = .386)	
	Indistinguishable	6 (4.1%)	-		-	
Max diameter (cm)	Mean ± SD	3.0 ± 1.5	1.14 (0.97–1.34, *p* = .100)		1.18 (1.01–1.38, *p* = .032)	1.00 (0.83–1.21, *p* = .989)
Smoking history	No	55 (37.7%)				
	Yes	91 (62.3%)	1.30 (0.79–2.15, *p* = .299)		1.54 (0.94–2.51, *p* = .085)	
Family history	N0	118 (62.8%)				
	Yes	28 (19.2%)	1.07 (0.60–1.92, *p* = .817)		1.07 (0.62–1.87, *p* = .798)	
Intraoperative blood loss (ml)	Mean ± SD	292.7 ± 282.1	1.00 (1.00–1.00, *p* = .021)	1.00 (1.00–1.00, *p* = .493)	1.00 (1.00–1.00, *p* = .031)	1.00 (1.00–1.00, *p* = .323)
Blood transfusion	No	109 (74.7%)				
	Yes	37 (25.3%)	1.30 (0.78–2.17, *p* = .312)		1.25 (0.76–2.05, *p* = .372)	
Approach	Thoracotomy	22 (15.1%)				
	VATS	116 (79.5%)	1.32 (0.65–2.68, *p* = .438)		1.17 (0.59–2.29, *p* = .653)	
	VATS converted to thoracotomy	8 (5.5%)	2.71 (0.91–8.11, *p* = .074)		2.58 (0.94–7.12, *p* = .067)	
Resection extent	Lobectomy	100(68.5%)				
	Combined lobectomy	36(24.7%)	1.27 (0.73–2.18, *p* = .397)		1.24 (0.74–2.09, *p* = .411)	
	Sublobectomy	10 (6.8%)	1.81 (0.77–4.24, *p* = .174)		1.88 (0.80–4.39, *p* = .148)	
T stage	1	35 (24.0%)				
	2	81 (55.5%)	2.24 (1.09–4.60, *p* = .028)	2.79 (1.30–6.00, *p* = .009)	2.00 (1.01–3.97, *p* = .048)	2.55 (1.18–5.52, *p* = .017)
	3	24 (16.4%)	2.44 (1.04–5.71, *p* = .040)	1.52 (0.58–3.95, *p* = .393)	2.24 (0.99–5.05, *p* = .052)	1.42 (0.52–3.85, *p* = .496)
	4	6 (4.1%)	8.64 (2.86–26.07, *p* < .001)	7.33 (2.08–25.80, *p* = .002)	11.36 (4.04–31.93, *p* < .001)	10.58 (3.06–36.61, *p* < .001)
N stage	0	52 (35.6%)				
	1	39 (26.7%)	3.98 (1.88–8.42, *p* < .001)	1.74 (0.52–5.86, *p* = .371)	3.47 (1.72–6.99, *p* < .001)	1.60 (0.46–5.59, *p* = .458)
	2	55 (37.7%)	6.09 (3.02–12.26, *p* < .001)	1.57 (0.40–6.19, *p* = .523)	4.82 (2.53–9.20, *p* < .001)	1.21 (0.30–4.91, *p* = .790)
M stage	0	139 (95.2%)				
	1	5 (3.4%)	1.55 (0.49–4.92, *p* = .461)		1.37 (0.43–4.37, *p* = .591)	
	2	2 (1.4%)	1.28 (0.18–9.21, *p* = .808)		1.04 (0.14–7.47, *p* = .972)	
TNM stage	1	46 (31.5%)				
	2	25 (17.1%)	3.94 (1.55–10.03, *p* = .004)	2.34 (0.57–9.58, *p* = .237)	3.25 (1.36–7.72, *p* = .008)	2.16 (0.53–8.88, *p* = .284)
	3	68 (46.6%)	7.73 (3.49–17.14, *p* < .001)	5.01 (1.04–24.22, *p* = .045)	6.04 (2.96–12.31, *p* < .001)	4.96 (1.05–23.38, *p* = .043)
	4	7 (4.8%)	25.11 (6.35–99.29, *p* < .001)	26.98 (4.87–149.50, *p* < .001)	15.16 (4.04–56.82, *p* < .001)	17.96 (3.67–87.88, *p* < .001)
Ki67 ≥ 60%	No	142 (97.3%)				
	Yes	4 (2.7%)	1.44 (0.35–6.00, *p* = .615)		0.98 (0.30–3.19, *p* = .974)	
Pre-op SCCAg high (> 2.7 ng/ml)	NO	7 (7.8%)				
	Yes	83 (92.2%)	0.50 (0.07–3.61, *p* = .492)		0.55 (0.08–3.95, *p* = .552)	
Pre-op CEA high (> 5.0 ng/ml)	No	132 (90.4%)				
	Yes	14 (9.6%)	2.50 (1.31–4.79, *p* = .006)	2.71 (1.36–5.39, *p* = .004)	2.40 (1.26–4.58, *p* = .008)	2.39 (1.21–4.72, *p* = .012)
Pre-op NSE high (> 16.3 ng/ml)	NO	107 (73.3%)				
	Yes	39 (26.7%)	1.13 (0.67–1.91, *p* = .634)		1.16 (0.70–1.93, *p* = .570)	
Pre-op Cyfra-211 high (> 3.5 ng/ml)	NO	130 (89.0%)				
	Yes	16 (11.0%)	1.27 (0.63–2.55, *p* = .509)		1.50 (0.79–2.85, *p* = .218)	
Pre-op proGRP high (> 69.2 ng/ml)	NO	98 (67.1%)				
	Yes	48 (32.9%)	1.27 (0.77–2.08, *p* = .346)		1.36 (0.84–2.21, *p* = .217)

**Table 4:** Univariable and multivariable analyses of disease-free survival and overall survival for all the enrolled LCNEC patients

Item		all	DFS		OS	
			HR (univariable)	HR (multivariable)	HR (univariable)	HR (multivariable)
Gender	Male	64 (82.1%)				
	Female	14 (17.9%)	0.71 (0.28–1.83, *p* = .482)		0.76 (0.32–1.81, *p* = .531)	
Age (years)	Mean ± SD	62.9 ± 9.2	1.02 (0.98–1.06, *p* = .311)		1.03 (0.99–1.07, *p* = .133)	
Neoadjuvant chemotherapy	No	70 (89.7%)				
	Yes	8 (10.3%)	2.15 (0.89–5.18, *p* = .090)		2.02 (0.84–4.85, *p* = .118)	
Adjuvant chemotherapy	No	19(24.36%)				
	Yes	59(75.64%)	22.00 (2.08–147.92, *p* = 1.02)		26.00(10.17–41.83, *p* = 0.212)	
Adjuvant radiotherapy	No	71 (91.0%)				
	Yes	7 (9.0%)	2.60 (1.08–6.26, *p* = .033)	1.23 (0.40–3.79, *p* = .712)	2.07 (0.86–4.96, *p* = .104)	
Duration of disease (months)	Mean ± SD	3.9 ± 4.1	1.00 (0.92–1.08, *p* = .952)		1.00 (0.93–1.07, *p* = .953)	
Symptoms	No	43 (55.1%)				
	Yes	35 (44.9%)	1.86 (0.98–3.53, *p* = .059)		1.73 (0.93–3.22, *p* = .084)	
History of other malignancies	No	76 (97.4%)				
	Yes	2 (2.6%)	-		23.51 (2.33–236.96, *p* = .007)	180.32 (11.60–2802.78, *p* < .001)
Comorbid cardiovascular diseases	No	49 (62.8%)				
	Yes	29 (37.2%)	1.10 (0.57–2.13, *p* = .776)		1.33 (0.71–2.51, *p* = .372)	
Comorbid neurological diseases	No	74 (94.9%)				
	Yes	4 (5.1%)	0.93 (0.22–3.88, *p* = .925)		0.88 (0.21–3.66, *p* = .862)	
Comorbid endocrine diseases	No	68 (87.2%)				
	Yes	10 (12.8%)	2.24 (0.98–5.14, *p* = .057)		2.50 (1.14–5.47, *p* = .022)	2.23 (0.84–5.93, *p* = .108)
Comorbid respiratory diseases	No	67 (85.9%)				
	Yes	11 (14.1%)	0.42 (0.13–1.38, *p* = .155)		0.60 (0.21–1.68, *p* = .332)	
Tumor location	Central	21 (26.9%)				
	Peripheral	57 (73.1%)	0.39 (0.20–0.75, *p* = .005)	0.65 (0.29–1.46, *p* = .298)	0.44 (0.23–0.83, *p* = .011)	0.54 (0.25–1.18, *p* = .122)
Max diameter (cm)	Mean ± SD	3.5 ± 2.2	1.36 (1.19–1.56, *p* < .001)	1.22 (0.89–1.67, *p* = .222)	1.33 (1.16–1.52, *p* < .001)	1.14 (0.88–1.48, *p* = .317)
Smoking history	No	18 (23.1%)				
	Yes	60 (76.9%)	1.03 (0.47–2.25, *p* = .938)		1.08 (0.51–2.27, *p* = .842)	
Family history	N0	69 (88.5%)				
	Yes	9 (11.5%)	0.39 (0.09–1.61, *p* = .192)		0.60 (0.18–1.93, *p* = .388)	
Intraoperative blood loss (ml)	Mean ± SD	296.7 ± 810.4	1.00 (1.00–1.00, *p* = .007)	1.00 (1.00–1.00, *p* = .558)	1.00 (1.00–1.00, *p* = .006)	1.00 (1.00–1.00, *p* = .151)
Blood transfusion	No	75 (96.2%)				
	Yes	3 (3.8%)	1.84 (0.44–7.68, *p* = .403)		1.72 (0.41–7.17, *p* = .455)	
Approach	Thoracotomy	33 (42.3%)				
	VATS	39 (50.0%)	0.19 (0.09–0.41, *p* < .001)	0.35 (0.14–0.89, *p* = .027)	0.27 (0.14–0.54, *p* < .001)	0.41 (0.17–0.99, *p* = .048)
	VATS converted to thoracotomy	6 (7.7%)	0.22 (0.05–0.91, *p* = .037)	0.19 (0.04–0.99, *p* = .049)	0.22 (0.05–0.94, *p* = .041)	0.20 (0.04–1.05, *p* = .057)
Resection extent	Lobectomy	56 (71.8%)				
	Combined lobectomy	11 (14.1%)	2.48 (1.11–5.53, *p* = .026)	1.81 (0.65–5.10, *p* = .259)	2.14 (0.97–4.75, *p* = .061)	
	Sublobectomy	11 (14.1%)	0.86 (0.33–2.25, *p* = .757)	1.67 (0.37–7.47, *p* = .504)	1.00 (0.41–2.44, *p* = .992)	
T stage	1	26(33.3%)				
	2	33(42.3%)	1.96(0.80,4.81, *p* = .142)	1.61 (0.40–6.50, *p* = .502)	2.00(1.01,3.97, *p* = 0.048)	1.15 (0.40–3.34, *p* = .797)
	3	13(16.7%)	4.55(1.72,12.01, *p* = .002)	1.27 (0.19–8.37, *p* = .802)	2.24(0.99,5.05, *p* = 0.052)	0.72 (0.14–3.66, *p* = .688)
	4	6(7.7%)	19.46(5.89,64.33, *p* < .001)	3.45 (0.37–32.09, *p* = .276)	11.36(4.04,31.93, *p* < .001)	2.69 (0.40–17.84, *p* = .306)
N stage	0	52(66.7%)				
	1	13(16.7%)	3.02(1.35,6.76, *p* = .007)	2.62 (0.73–9.42, *p* = .139)	3.47(1.72,6.99, *p* < .001)	1.85 (0.53–6.46, *p* = .334)
	2	13(16.7%)	5.72(2.63,12.44, *p* < .001)	0.33 (0.04–2.46, *p* = .280)	4.82(2.53,9.20, *p* < .001)	0.25 (0.03–1.85, *p* = .176)
M stage	0	74(94.9%)				
	1	4(5.1%)	1.27(0.31,5.30,*p* = .742)		1.37(0.42,4.37, *p* = .591)	
	2	0	-		1.04(0.14,7.47, *p* = .972)	
TNM stage	1	37 (47.4%)				
	2	19 (24.4%)	2.57 (1.07–6.17, *p* = .035)	0.80 (0.21–3.03, *p* = .747)	2.59 (1.11–6.02, *p* = .027)	1.38 (0.38–5.08, *p* = .626)
	3	18 (23.1%)	10.41 (4.53–23.94, *p* < .001)	6.73 (0.81–55.93, *p* = .078)	9.53 (4.23–21.47, *p* < .001)	8.72 (1.03–73.70, *p* = .047)
	4	4 (5.1%)	5.84 (1.26–27.09, *p* = .024)	13.66 (2.23–83.72, *p* = .005)	6.10 (1.31–28.43, *p* = .021)	14.01(2.49–78.75, *p* = .003)
Ki67 ≥ 60%	No	10(20%)				
	Yes	40(80%)	1.02(0.37,2.79,*p* = .971)		0.98(0.30,3.19, *p* = .974)	
Pre-op SCCAg high (> 2.7 ng/ml)	NO	77 (98.7%)				
	Yes	1 (1.3%)	3.21 (0.43–23.96, *p* = .255)		2.00 (0.27–14.71, *p* = .495)	
Pre-op CEA high (> 5.0 ng/ml)	No	62 (79.5%)				
	Yes	16 (20.5%)	1.09 (0.51–2.30, *p* = .829)		1.09 (0.51–2.29, *p* = .829)	
Pre-op NSE high (> 16.3 ng/ml)	NO	61 (78.2%)				
	Yes	17 (21.8%)	1.38 (0.67–2.84, *p* = .385)		1.30 (0.63–2.66, *p* = .477)	
Pre-op Cyfra-211 high (> 3.5 ng/ml)	NO	69 (88.5%)				
	Yes	9 (11.5%)	1.35 (0.52–3.46, *p* = .536)		1.46 (0.61–3.47, *p* = .393)	
Pre-op proGRP high (> 69.2 ng/ml)	NO	68 (87.2%)				
	Yes	10 (12.8%)	0.46 (0.14–1.51, *p* = .200)		0.48 (0.15–1.55, *p* = .220)	

Turning to the multivariate analysis of OS, increasing age at diagnosis emerged as an independent risk factor (HR 1.03, 95% CI 1.01–1.06, *p* = 0.002). As with DFS, undergoing VATS surgery was a powerful predictor of improved OS (HR 0.49, 95% CI 0.33–0.74, *p* = 0.001). Consistent with the DFS findings, advancing T stage corresponded with poorer OS, as patients with T2 (HR 1.64, 95% CI 1.01–2.65, *p* = 0.044) and T4 (HR 3.20, 95% CI 1.37–7.51, *p* = 0.007) lesions exhibited poorer prognosis1. Similarly, advancing TNM stage emerged as a potent predictor for unfavorable prognosis (Table 2).

Within the SCLC subgroup, advancing T stage continued to predict poorer OS, and advancing TNM stage also emerged as a profound predictor. Additionally, elevated preoperative CEA levels independently predicted poorer OS (HR 2.39, 95% CI 1.21–4.72, *p* = 0.012) (Table 3). In the LCNEC subgroup, the presence of history of other malignancies emerged as an extraordinary adverse prognostic factor (HR 180.32, 95% CI 11.60–2802.78, *p* < 0.001). However, undergoing VATS approach conferred a significant OS benefit (HR 0.41, 95% CI 0.17–0.99, *p* = 0.048). Advancing to stage III and IV disease also emerged as profound predictors (Table 4). Notably, intraoperative blood loss emerged as an adverse prognostic factor in univariate OS analysis for the overall cohort and both subgroups. However, as the analysis results display only four decimal places, the precise hazard ratios could not be reported. In the multivariate OS analysis for the overall cohort, each milliliter of intraoperative blood loss was associated with a marginal 0.03% increased risk of death (multivariable HR 1.0003, 95% CI 1.0001–1.0005, *p* = 0.014).

### Nomogram model and model verification

**Fig. 3:**
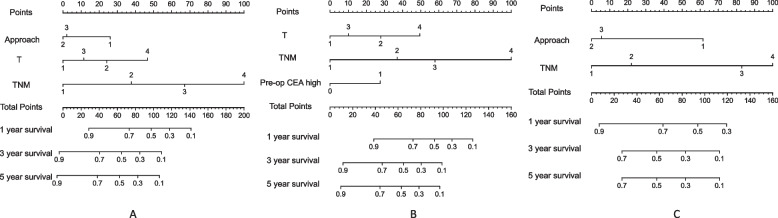
Nomogram model predicting the 1-, 3- and 5-year DFS in included patients. The nomogram is used by summing all points identified on the scale for each variable. The total points projected on the bottom scales indicate the probabilities of 1-, 3- and 5-year survival: **A** pHGNEC; **B** SCLC; **C** LCNEC

**Fig. 4:**
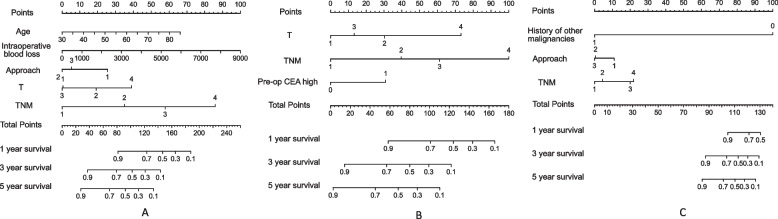
Nomogram model predicting the 1-, 3- and 5-year OS in included patients. The nomogram is used by summing all points identified on the scale for each variable. The total points projected on the bottom scales indicate the probabilities of 1-, 3- and 5-year survival: **A** pHGNEC; **B** SCLC; **C**. LCNEC

**Fig. 5:**
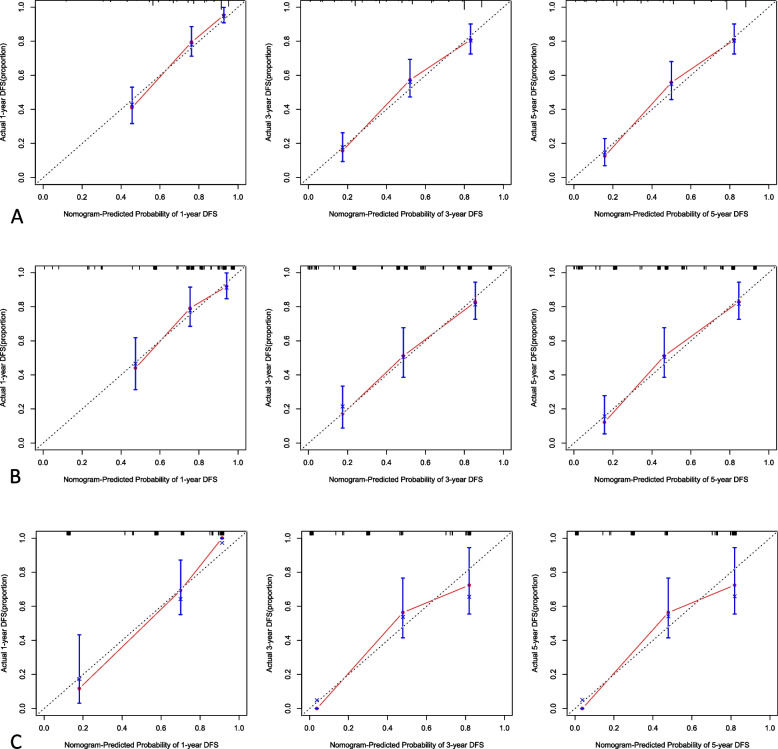
The calibration curves for predicting patient DFS at 1-, 3- and 5-year in the internal verification: **A** pHGNEC; **B** SCLC; **C** LCNEC. The DFS predicted by the nomogram model is plotted on the x-axis, and the actual DFS is plotted on the y-axis

**Fig. 6:**
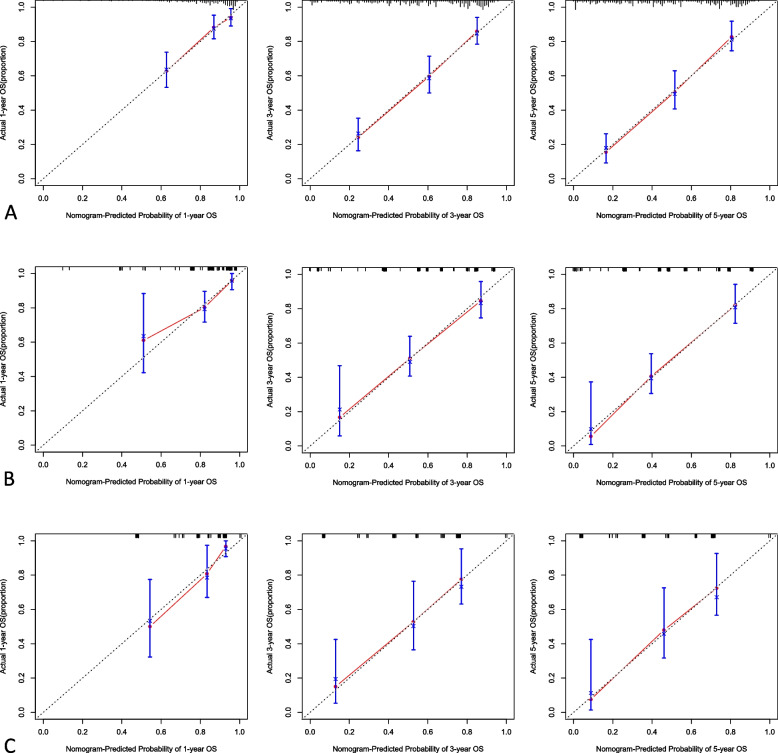
The calibration curves for predicting patient OS at 1-, 3- and 5-year in the internal verification: **A** pHGNEC; **B** SCLC; **C** LCNEC. The OS predicted by the nomogram model is plotted on the x-axis, and the actual OS is plotted on the y-axis

Nomogram model that included the important predictors in the Cox analysis was established to predict the prognosis of pHGNEC, including SCLC and LCNEC (Figs. 3 and 4). Internal verification also showed that the nomogram could accurately predict the C-index of DFS for included pHGNEC, SCLC and LCNEC, which was 0.757, 0.756 and 0.793. Internal verification showed that the nomogram could accurately predict the C-index of OS for included pHGNEC, SCLC and LCNEC, which was 0.748, 0.741 and 0.787. The calibration curve showed that there was good concordance between the predicted and observed values of 1-year and 3-year OS and DFS internal validation cohorts (Figs. 5 and 6).

## Discussion

The prognostic factors of pHGNEC have been extensively studied in recent years. This study retrospectively analyzed the prognosis and clinicopathological data of patients with resected pHGNEC at our institution to identify factors impacting postoperative outcomes. We developed a nomogram to visualize study findings and provide meaningful references to guide individualized treatment.

The role of preoperative biopsy in managing HGNEC is controversial. While some studies show preoperative biopsy can accurately determine grade and guide surgery, others demonstrate high sampling error rates and inaccurate grading leading to undertreatment [11]. Given the aggressive HGNEC behavior, reliance on preoperative biopsy alone risks misclassification and inappropriate management. In our study, 75 enrolled patients underwent preoperative bronchoscopic or CT-guided biopsy, but only 34 yielded results consistent with final pathology – an accuracy under 50%. As reported, the complex neuroendocrine pathology makes small sample biopsy morphologically difficult to interpret [12]. Discrepancies between preoperative and final pathology are also not uncommon [13, 14]. Current literature suggests that the accuracy of intraoperative frozen section pathology in diagnosing pHGNEC is less than ideal [15]. Similarly, our study showed intraoperative frozen section diagnosis accuracy was suboptimal at under 50% (81/169). Despite limitations in evaluating mitoses and invasion, frozen section analysis of the whole tumor enables optimized surgical decision-making. Thus, the diagnostic accuracy of preoperative and intraoperative pHGNEC pathology remains controversial. Relying solely on limited sampling risks significant errors. Beyond treatment, surgery provides invaluable pHGNEC diagnosis not afforded by other modalities.

Approximately two-thirds of SCLC and 40–70% of LCNEC cases present as extensive stage or stage III-IV disease at diagnosis [16–18]. After resection of pHGNEC, prognosis is linked to tumor size, invasion, and TNM stage. Larger tumors with higher T-status (indicating more invasion) and occult metastases lead to poorer survival. T1 tumors (limited to lung) fare better than T2 (invading pleura or bronchi), and T3-T4 (invading chest wall or mediastinum) are worse. TNM stage also considers nodal and distant spread, with stage I/II showing the best prognosis. In our study, stage III/IV HGNEC patients had worse survival than stage I/II in both SCLC and LCNEC subgroups. A Surveillance, Epidemiology, and End Results (SEER) database study of stage III-IV LCNEC and SCLC also found TNM stage correlated with prognosis [16–18]. However, selected stage III patients may benefit from aggressive debulking. Disseminated stage IV disease is a contraindication to resection given dismal outcomes.

The optimal surgical strategy for resectable pHGNEC remains debated. Both extent of resection and technique influence postoperative complications, mortality, and long-term prognosis. Compared to open thoracotomy, VATS lobectomy demonstrates reduced pain, shorter hospitalization, quicker recovery, and fewer complications [19]. Recent small series show equivalent oncologic resection with lower morbidity, suggesting minimally invasive approaches do not compromise survival or recurrence [20]. In our study, VATS was associated with better prognosis, reflecting high resection eligibility. Several studies report lobectomy achieves better long-term outcomes than limited resection [21–23]. The study covered a long period, during which our surgical methods shifted from open chest to less invasive procedures. Some patients' surgery types were chosen based on these changes. Surgeons' expertise also played a part, with thoracotomy sometimes preferred for advanced pHGNEC cases to ensure a safer and smoother operation. The feasibility of extensive resection depends on locoregional findings. Achieving microscopically negative margins is essential, with completeness of resection among the strongest positive prognostic indicators for these aggressive tumors. A study by Haruki et al. found lobectomy or more plus mediastinal lymph node dissection and adjuvant chemotherapy provided better pHGNEC outcomes than limited treatment [24]. However, we found worse prognosis for patients undergoing complex or combined lobectomies. Pursuing R0 resection may necessitate extended surgery, reflecting advanced local invasion and explaining worse outcomes.

Lung cancer prognosis is complex, influenced by factors such as cancer type, stage at diagnosis, patient characteristics, and treatment approaches. While advances in screening, diagnosis, and treatments like targeted therapies and immune checkpoint inhibitors have improved survival for some NSCLC and SCLC patients, lung cancer remains a leading cause of cancer-related deaths globally, with an estimated 2 million new cases and 1.76 million deaths annually [25]. Additionally, a history of previous malignancies may impact lung cancer prognosis, with some studies suggesting prior cancer history does not reduce survival in early-stage, locally advanced, or advanced lung cancer, [26, 27] while others note a prognostic effect [28]. Our study found that while a history of previous malignant tumors did not significantly impact DFS in patients with pHGNEC and LCNEC who underwent surgical treatment, it did predict a worse OS.

Two randomized trials evaluating surgical resection for limited stage small cell lung cancer failed to demonstrate a survival benefit with surgery [29, 30]. The role for operating on SCLC remains controversial given its aggressive course. To date, there is no evidence supporting surgical indication in stage II and stage IIIA SCLC. National Comprehensive Cancer Network (NCCN) guidelines, in fact, do not recommend resecting advanced tumors as they do not benefit from surgery, [10] although some recent reports seem to disclose a significant improvement in survival in stage II and stage IIIA SCLC undergoing lung resection [31]. Previous studies conducted at our institution have also validated this observation [32]. Identifying prognostic factors is therefore critical to guide management. Our study reveals elevated preoperative CEA independently associates with worse SCLC prognosis after resection. This suggests occult biological aggression warranting adjuvant chemotherapy consideration. Prior studies demonstrate links between CEA and non-small cell lung cancer (NSCLC) recurrence, mutations, and chemotherapy response [33–35]. While the mechanisms linking CEA to accelerated progression are unclear, possibilities include aberrant glycoprotein metabolism or epidermal growth factor receptor pathway activation promoting invasion. Regardless, this serum biomarker can better predict surgical futility and need for multimodality SCLC therapy.

The nomogram model presented in this study serves as a valuable tool for predicting the survival outcomes of patients with pHGNEC, encompassing both SCLC and LCNEC. The model integrates a multitude of prognostic factors, including surgical approach, tumor staging, age, and preoperative CEA levels, providing clinicians with a user-friendly interface to assess patient risk profiles. In the development of our nomograms, we have assumed linear relationships between the predictors and the outcomes. However, it is important to acknowledge that in clinical practice and potentially within the context of our study, complex interactions and nonlinear effects may be present, which could introduce bias into the predictions made by the nomograms. Consequently, we caution that the findings from our research should be interpreted as contributing to the hypothesis-building process rather than providing definitive, universally applicable conclusions.

The internal validation of the model demonstrates a commendable concordance index (C-index), indicating high predictive accuracy for disease-free survival (DFS) and overall survival (OS). However, the model's limitations, such as the absence of external validation and molecular profiling data, suggest the need for further development. Expanding the dataset to include diverse patient populations and incorporating additional biomarkers and molecular information could enhance the model's generalizability and precision. The nomogram's utility in guiding personalized treatment strategies is evident, with its potential to inform more aggressive therapeutic approaches for patients with poor prognoses and conservative management for those with favorable outcomes. The model's interpretability is crucial for both clinicians and patients, and efforts to improve its transparency are warranted. Regular updates and maintenance of the nomogram are essential to ensure its predictive capabilities remain current with advancing medical research and accumulating evidence.

This study, conducted as a retrospective single-institution analysis, is subject to several limitations that may affect the generalizability of its findings. The inherent selection biases, stemming from our center's specialization in managing complex cases, may not fully represent the broader patient population. The study's relatively small sample size, especially for certain segments of the analysis, necessitates cautious interpretation of the results and underscores the need for further research with larger cohorts. Additionally, the nomogram tool developed in this study has not been externally validated, which is crucial for ensuring its predictive accuracy across diverse patient populations. The absence of molecular profiling data in the study cohort also limits the integration of genetic markers with clinical variables, which could potentially enhance the prognostic models by providing a more comprehensive understanding of patient outcomes.

## Conclusion

In conclusion, this study identified minimally invasive surgery, younger age, early TNM stage, and absence of prior malignancy as independent favorable prognostic factors for resected pHGNEC. We also revealed preoperative CEA as a marker for increased risk predicting worse prognosis in resected SCLC patients specifically. Our study demonstrates the vital diagnostic role for surgery in analyzing whole tumor pathology unavailable by limited preoperative sampling. These prognostic factors and nomogram tool provide clinically applicable models to risk stratify patients, guide individualized treatment decisions, and warrant further research optimizing selection criteria for this aggressive disease.

## Abbreviations

pHGNEC: Pulmonary high-grade neuroendocrine carcinoma
SCLC: Small cell lung cancer
LCNEC: Large cell neuroendocrine carcinoma
OS: Overall survival
DFS: Disease-free survival
HR: Hazard ratios
CI: Confidence interval
VATS: Video-assisted thoracic surgery
CEA: Carcinoembryonic antigen
SEER: Surveillance, Epidemiology, and End Results
NCCN: National Comprehensive Cancer Network
NSCLC: Non-small cell lung cancer

## Declarations

### Availability of data and materials

The data that support the findings of this study are not openly available due to reasons of sensitivity and are available from the corresponding author upon reasonable request.
